# Supplementary material for: Hydroxylated Coumarin-Based Thiosemicarbazones as Dual Antityrosinase and Antioxidant Agents
Source: Int J Mol Sci. 2023 Jan 14;24(2):1678. doi: 10.3390/ijms24021678 (PMC9861830; doi:10.3390/ijms24021678)
Supplement: Supplementary file 1 [file ijms-24-01678-s001.zip › ijms-2129956-supplementary.pdf]

Hydroxylated Coumarin-Based Thiosemicarbazones as Dual Antityrosinase and Antioxidant Agents

Sebastiano Masuri [1,†], Benedetta Era [2,†], Francesca Pintus [2], Enzo Cadoni [1], Maria Grazia Cabiddu [1], Antonella Fais [2,*] and Tiziana Pivetta [1,*]

[1] Department of Chemical and Geological Sciences, University of Cagliari, S.P. 8 km 0.700, 09042 Cagliari, Italy
[2] Department of Life and Environmental Sciences, University of Cagliari, S.P. 8 km 0.700, 09042 Cagliari, Italy
* Correspondence: fais@unica.it (A.F.); tpivetta@unica.it (T.P.)
† These authors contributed equally to this work.

SUPPLEMENTARY INFORMATION

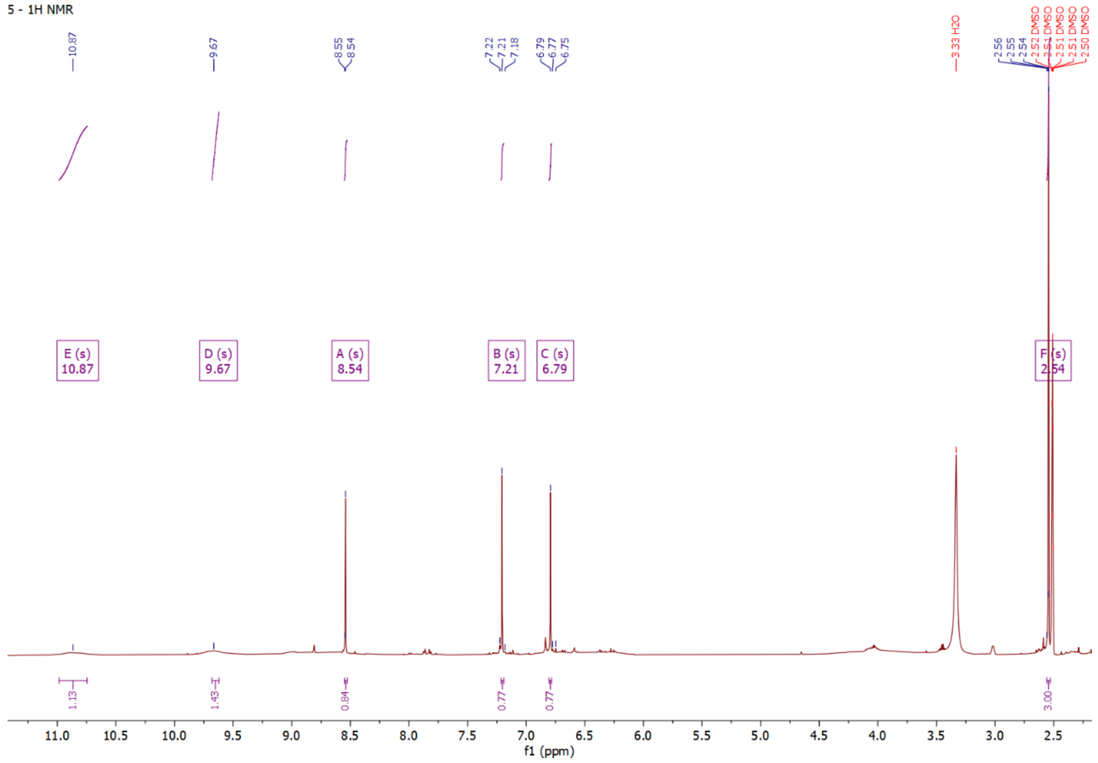

Figure S1. ^{1}H-NMR of **5**, 6,7-dihydroxy-3-acetyl-2*H*-chromen-2-one (600 MHz, DMSO d6).

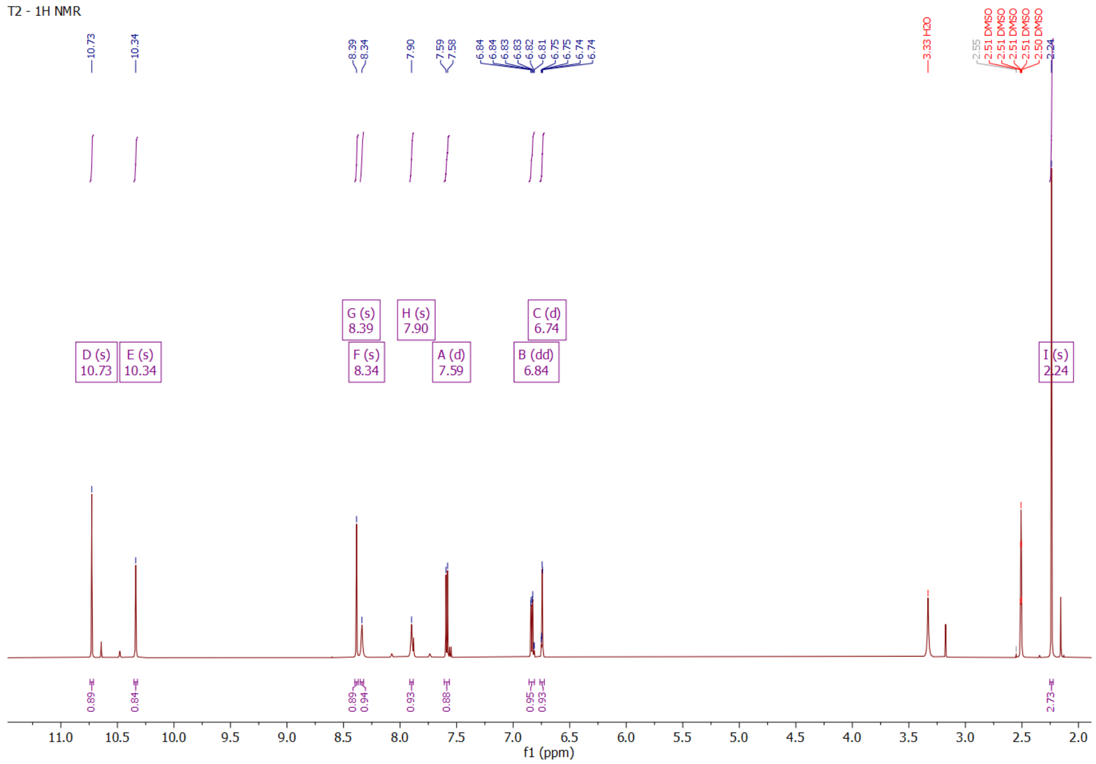

Figure S2. ^{1}H-NMR of **T2**, (1*E*)-2-(1-(6-hydroxy-2-oxo-2*H*-chromen-3-yl)ethylidene)hydrazine-1-carbothioamide (600 MHz, DMSO d6).

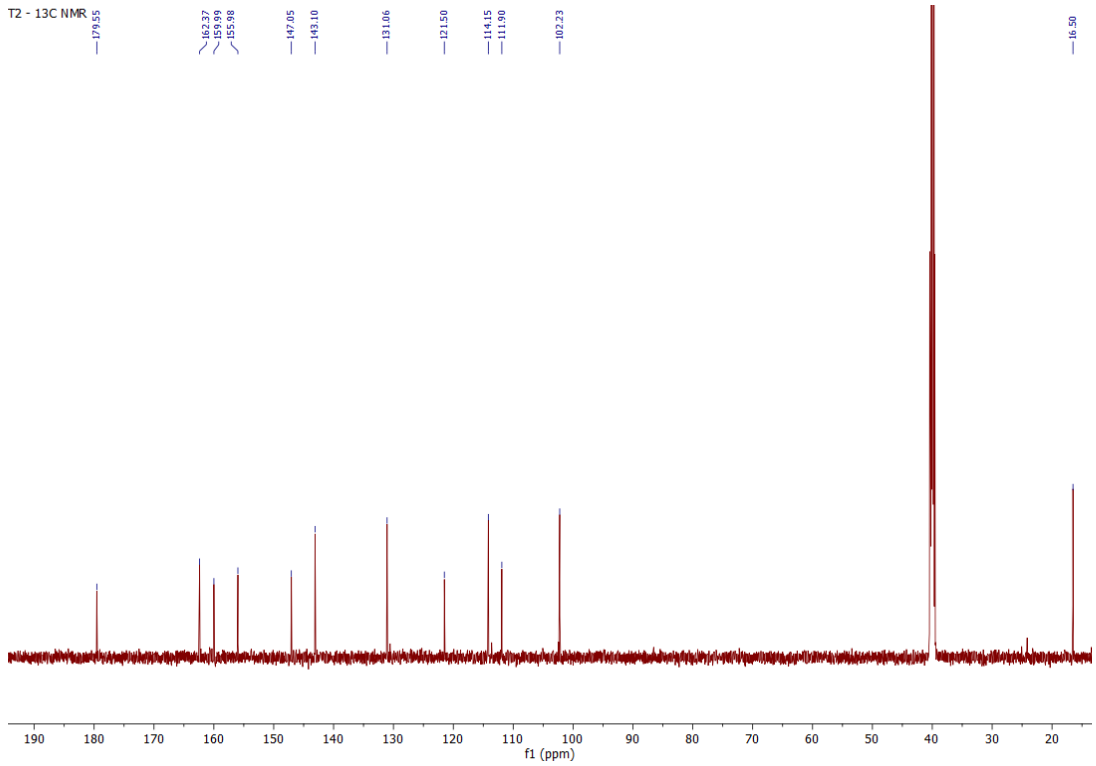

Figure S3. ^{13}C-NMR of **T2**, (1*E*)-2-(1-(6-hydroxy-2-oxo-2*H*-chromen-3-yl)ethylidene)hydrazine-1-carbothioamide (151 MHz, DMSO d6).

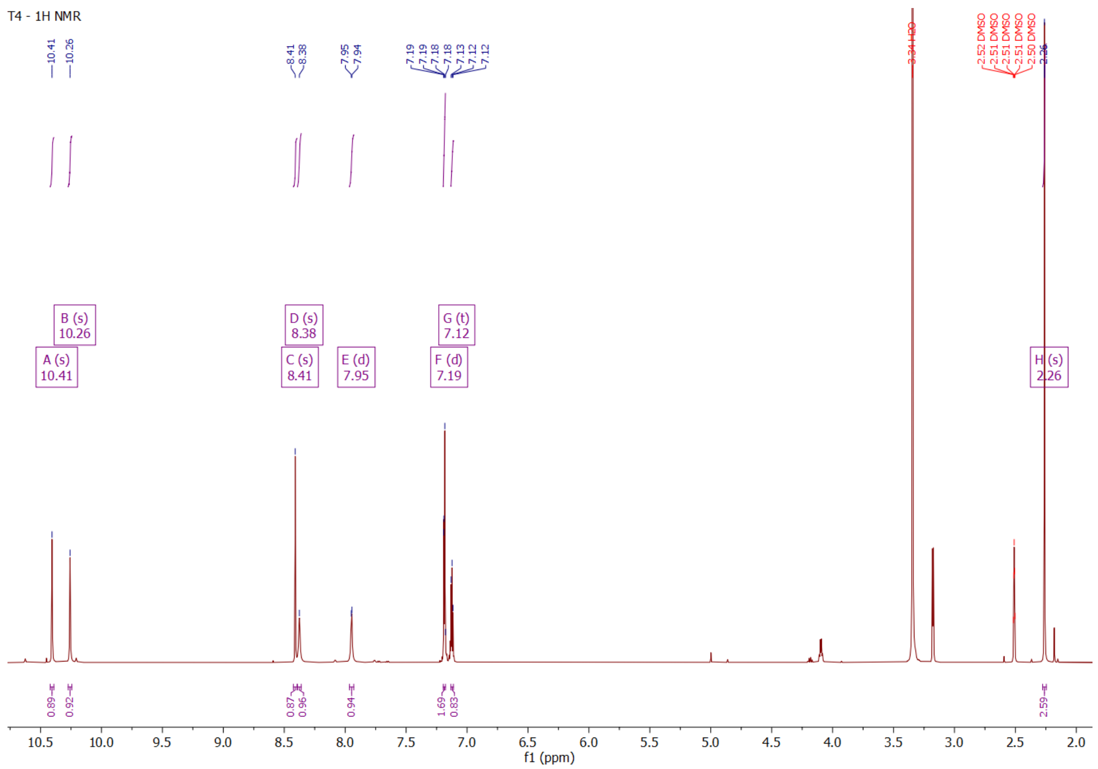

Figure S4. ^{1}H-NMR of **T4,** (1*E*)-2-(1-(8-hydroxy-2-oxo-2*H*-chromen-3-yl)ethylidene)hydrazine-1-carbothioamide (600 MHz, DMSO d6).

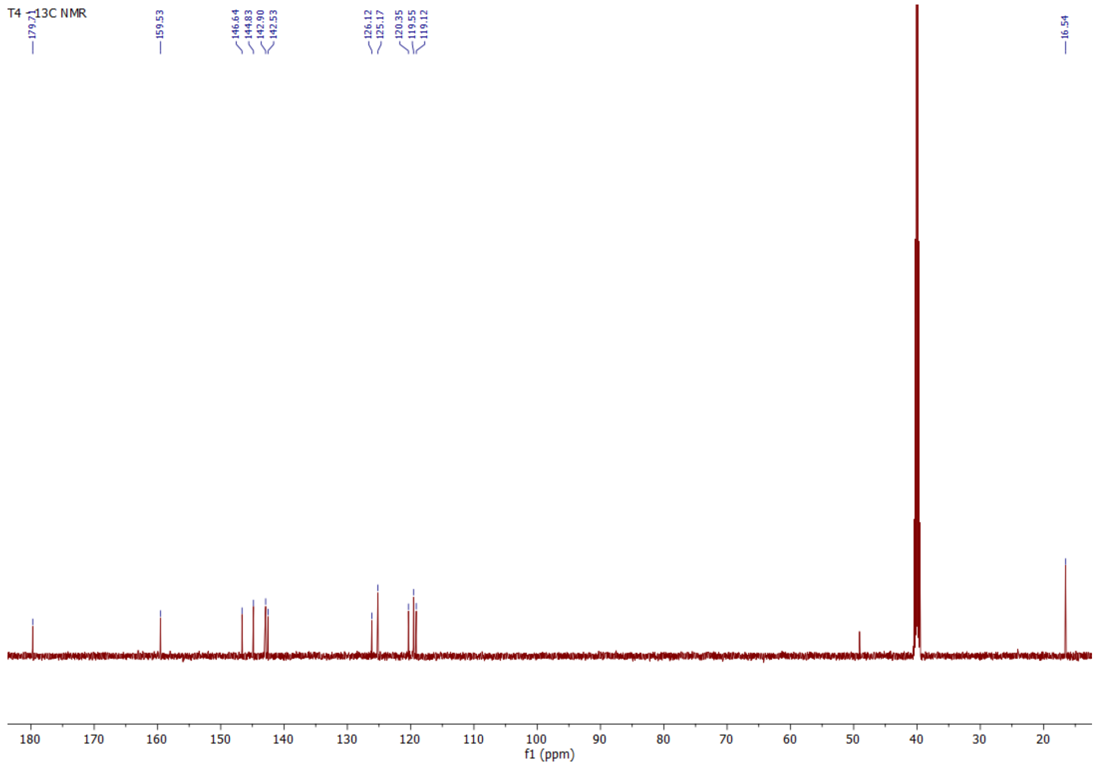

Figure S5. ^{13}C-NMR of **T4,** (1*E*)-2-(1-(8-hydroxy-2-oxo-2*H*-chromen-3-yl)ethylidene)hydrazine-1-carbothioamide (151 MHz, DMSO d6).

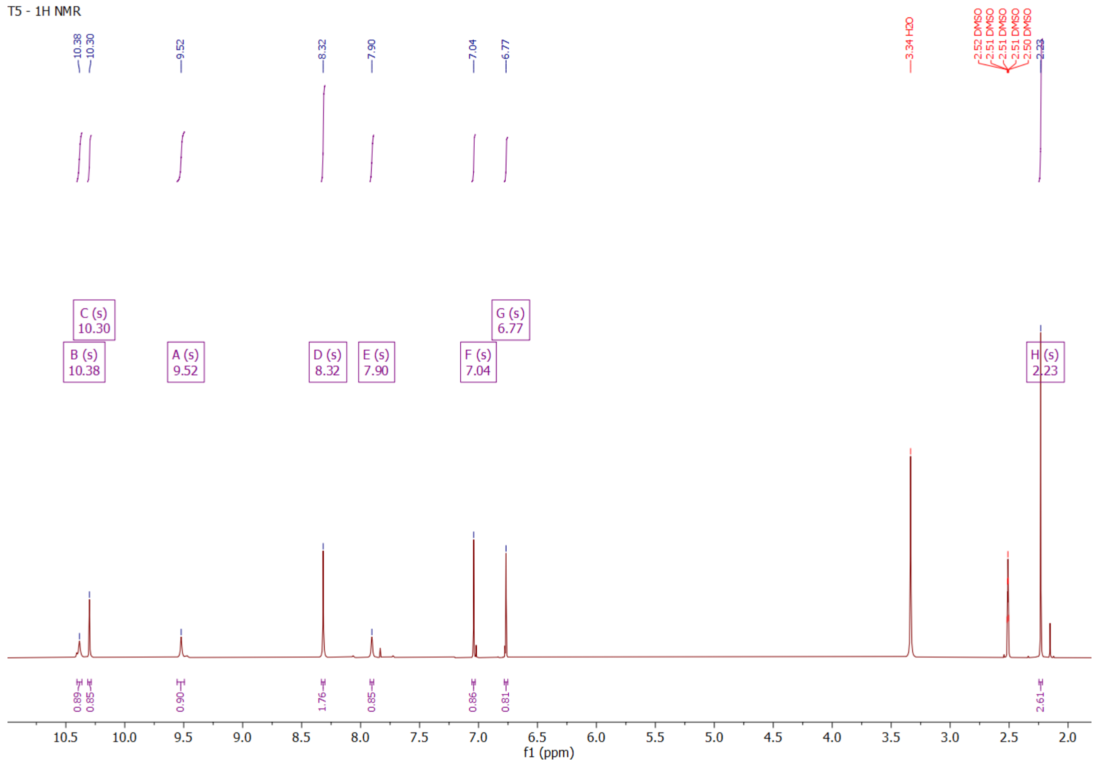

Figure S6. ^{1}H-NMR of **T5**, (1*E*)-2-(1-(6,7-dihydroxy-2-oxo-2*H*-chromen-3-yl)ethylidene)hydrazine-1-carbothioamide (600 MHz, DMSO d6).

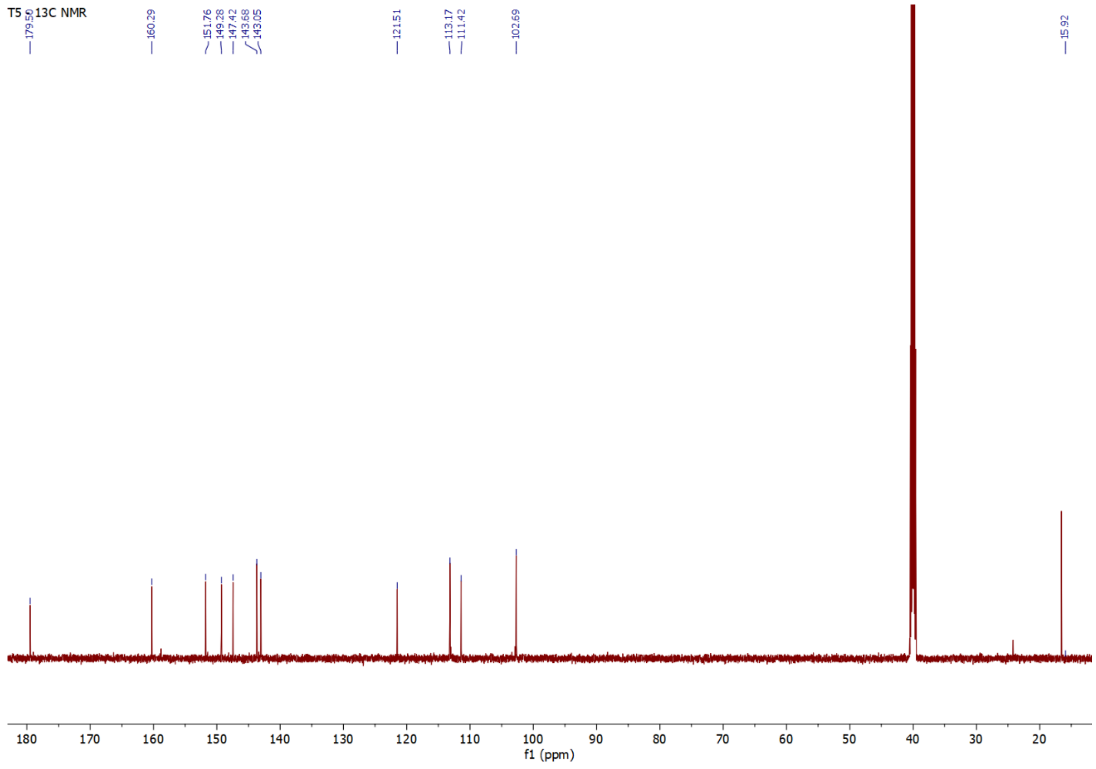

Figure S7. ^{13}C-NMR of **T5**, (1*E*)-2-(1-(6,7-dihydroxy-2-oxo-2*H*-chromen-3-yl)ethylidene)hydrazine-1-carbothioamide (151 MHz, DMSO d6).

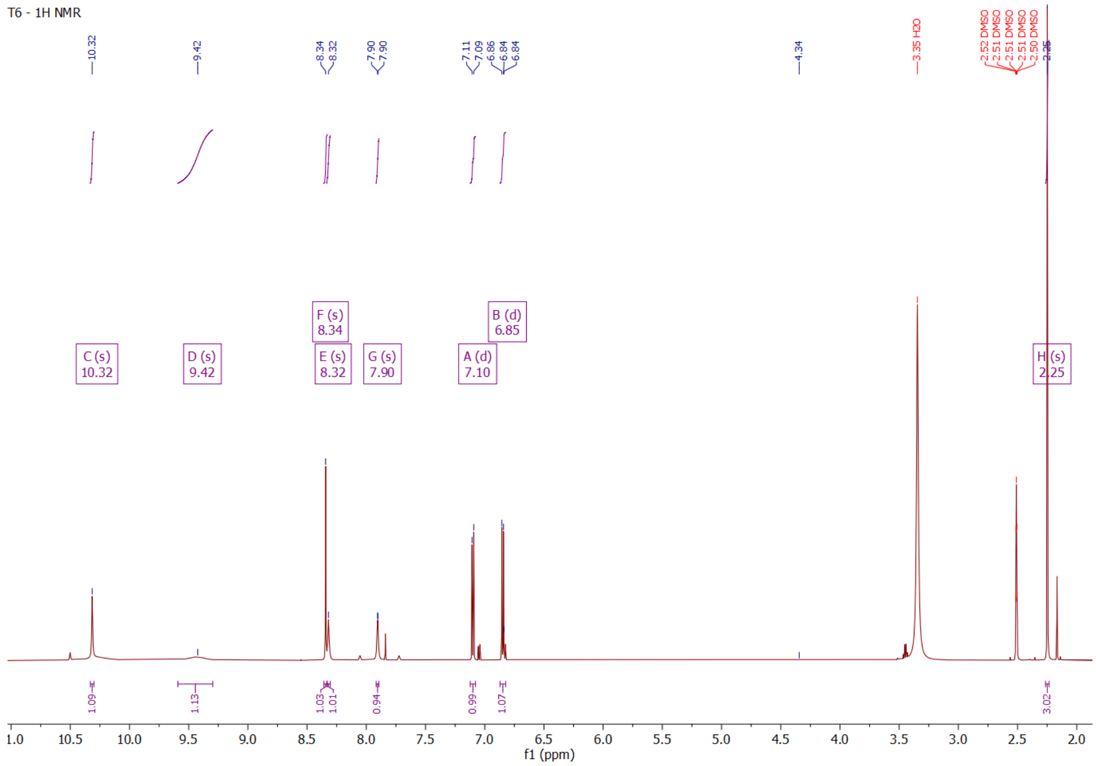

Figure S8. ^{1}H-NMR of **T6,** (1*E*)-2-(1-(7,8-dihydroxy-2-oxo-2*H*-chromen-3-yl)ethylidene)hydrazine-1-carbothioamide (600 MHz, DMSO d6).

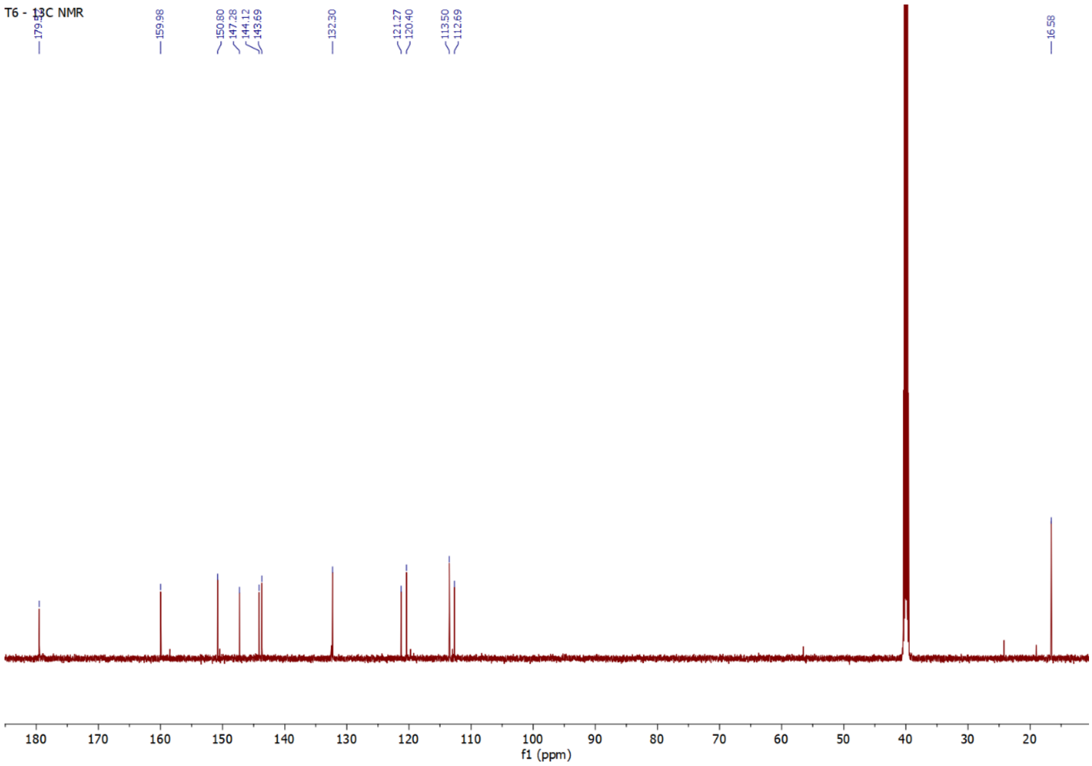

Figure S9. ^{13}C-NMR of **T6,** (1*E*)-2-(1-(7,8-dihydroxy-2-oxo-2*H*-chromen-3-yl)ethylidene)hydrazine-1-carbothioamide (151 MHz, DMSO d6).

A

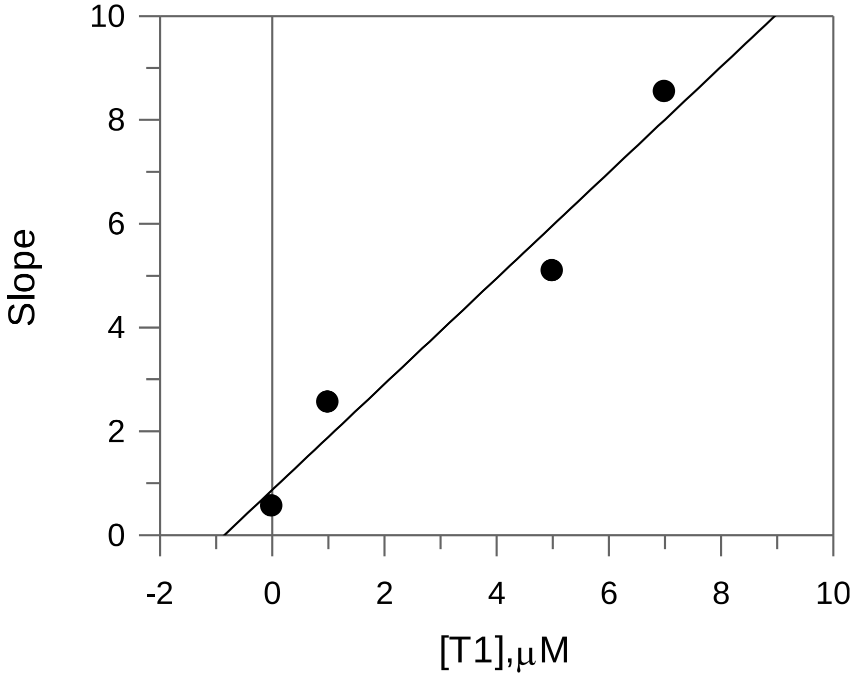

B

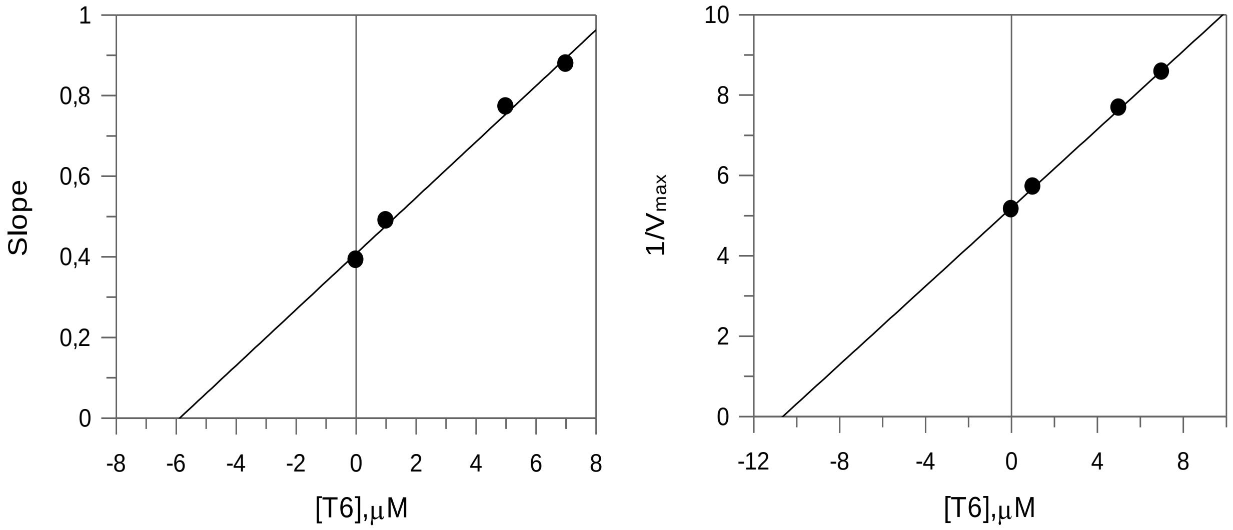

Figure S10. The secondary plot of slope versus concentration of compound **T1** (A) and **T6** (B), to determine the inhibition constant (Ki) and the secondary plot of 1/Vmax versus concentration of compound **T6** to determine the inhibition constant (Kis).

Spectral variations observed during the titration of T1

Four equilibria were evidenced during the titration with NaOH. At pH 3.33 two partially overlapped bands at 267 and 331 nm were present. Upon addition of base, a decrease in absorbance and the formation of an isosbestic point at 384 nm were observed. At pH 6.10, a blue shift (from 331 to 323 nm) was evidenced (**Figure S11A**). From pH 9.93 to 10.79, the decrease in absorbance at 271 nm was accompained by the formation of an isosbestic point at 284 nm and a change in the spectral shape in the 310-360 nm region (two overlapped peaks appeared at 313 and 359 nm, **Figure S11B**).

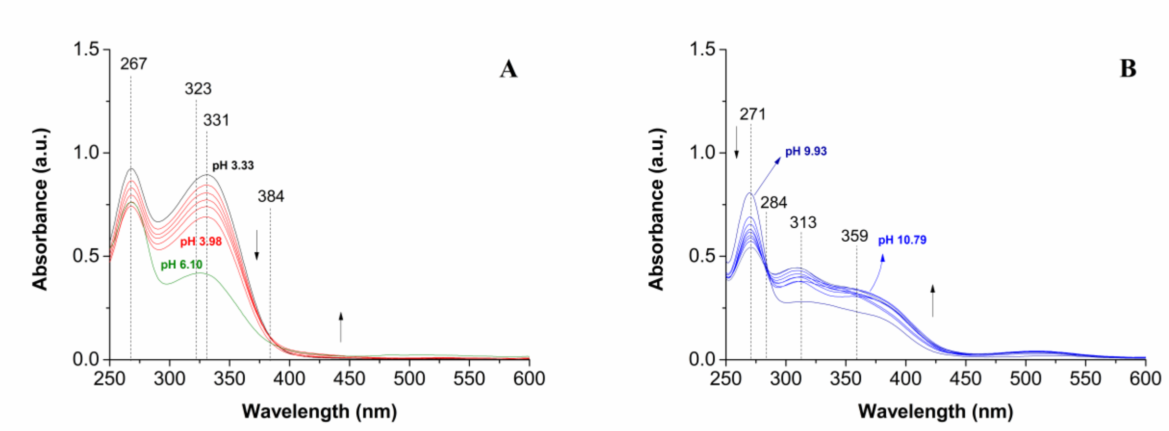

Figure S11. Selected spectra collected during the potentiometric and spectrophotometric titration of **T1** ($1.21 \cdot 10^{-4}$ M, 0.1 M NaCl, 25 °C, 0.5 cm optic path length) from pH 3.32 to 6.10 (**A**); from pH 9.93 to 10.79 (**B**).

Spectral variations observed during the titration of T5

The analysis of the spectra recorded during the titration with **T5** evidenced the presence of six equilibria. At pH 3.14 two bands at 268 and 370 nm were osberved, while a decrease in absorbance and the formation of an isosbsetic point at 390 nm is evidenced upon base addition. A small red shift (from 370 to 374 nm) and the formation of a shoulder at 434 nm were observed (**Figure S12A**). At pH 6.28 a new band at 418 nm was put into evidence,

while at pH 8.27 a red shift at 424 nm with the formation of a shoulder at 492 nm was observed (**Figure S12B**). In the pH range 9.66-10.78 the increase in absorbance at 425 nm and the formation of a shoulder at 483 nm are accompained by the formation of an isosbestic point at 372 nm (**Figure S12C**).

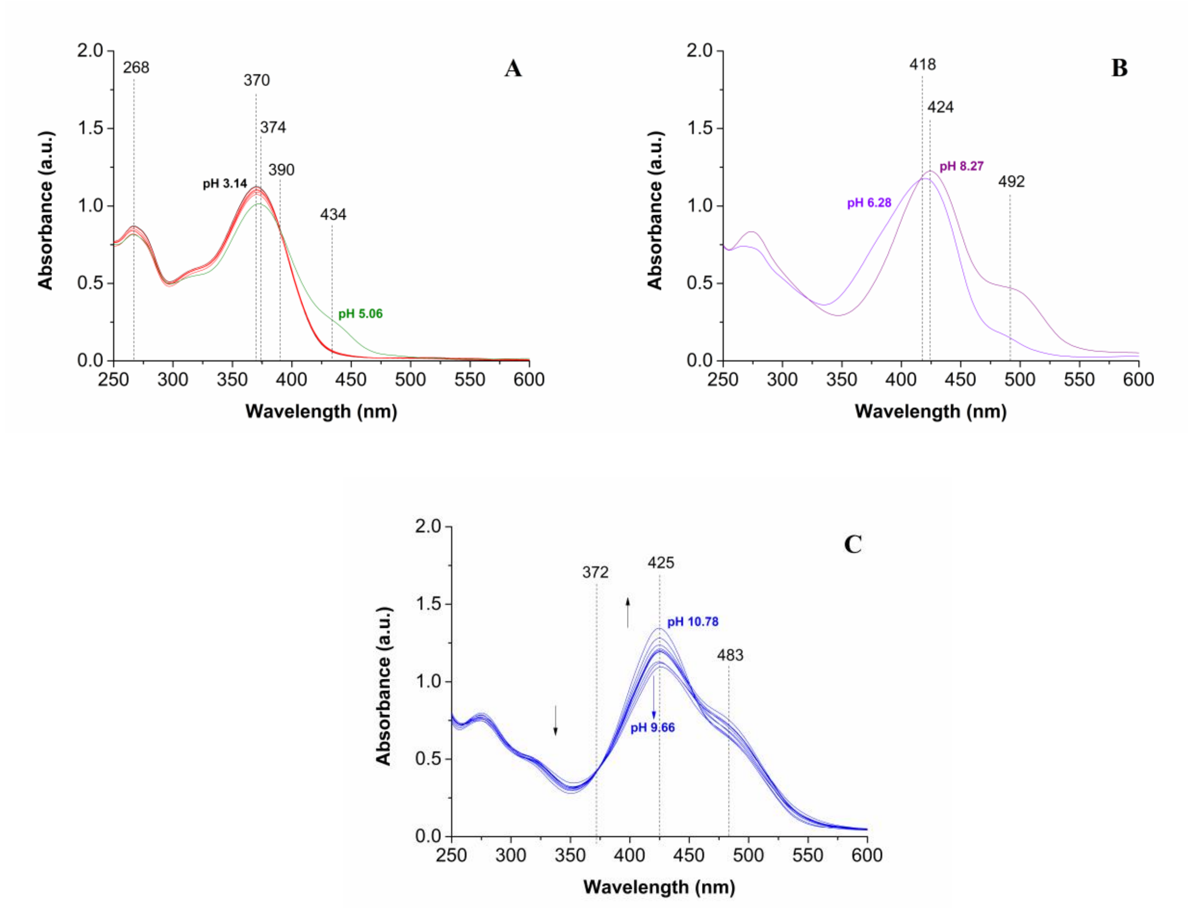

Figure S12. Selected spectra collected during the potentiometric and spectrophotometric titration of **T5** ($1.19 \cdot 10^{-4}$ M, 0.1 M NaCl, 25 °C, 0.5 cm optic path length) from pH 3.14 to 5.06 (**A**); from pH 6.28 to 8.27 (**B**); from pH 9.66 to 10.78 (**C**).

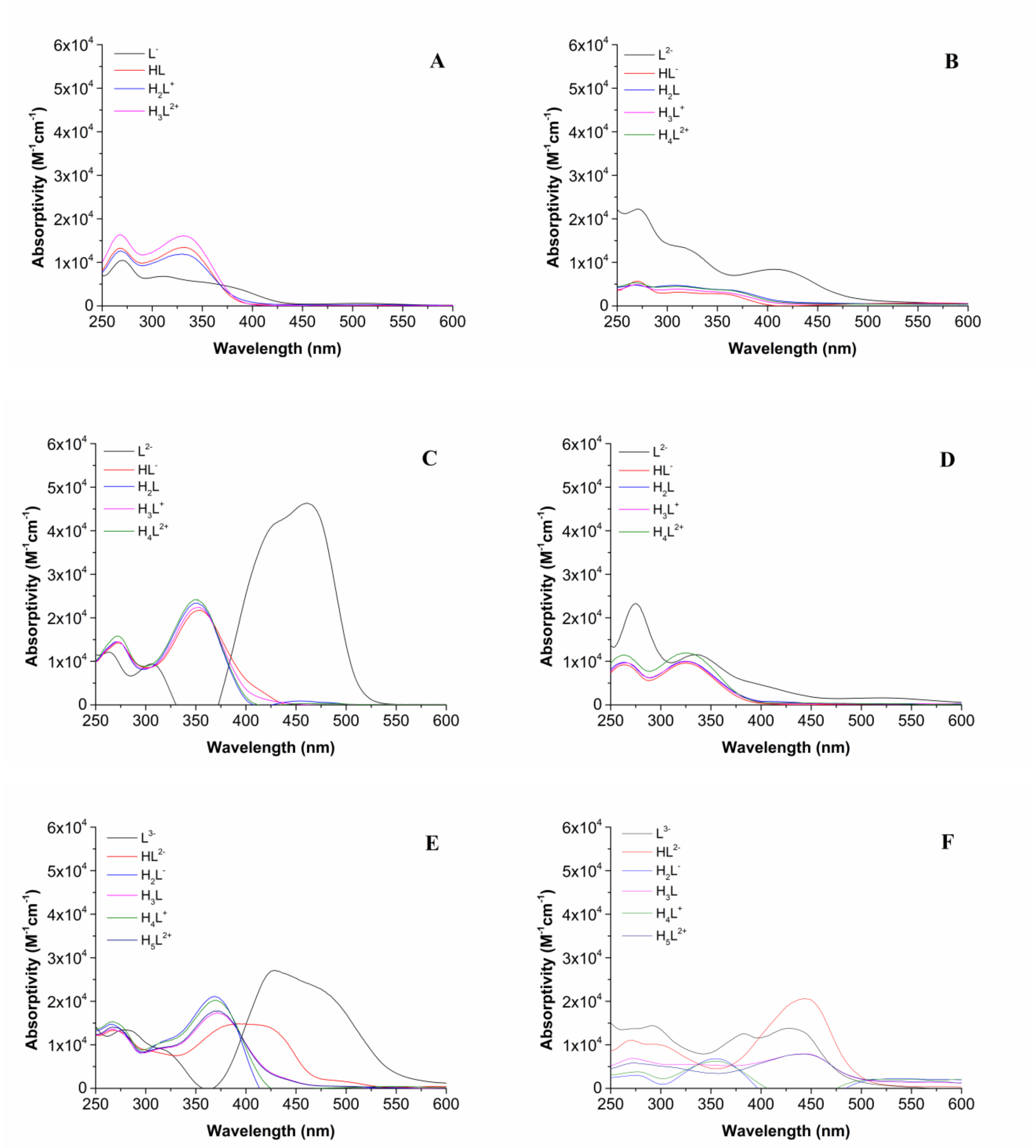

Figure S13. Calculated pure spectra obtained from the eigenvalue analyses of potentiometric and spectrophotometric titrations of **T1-6** (**A-F**).

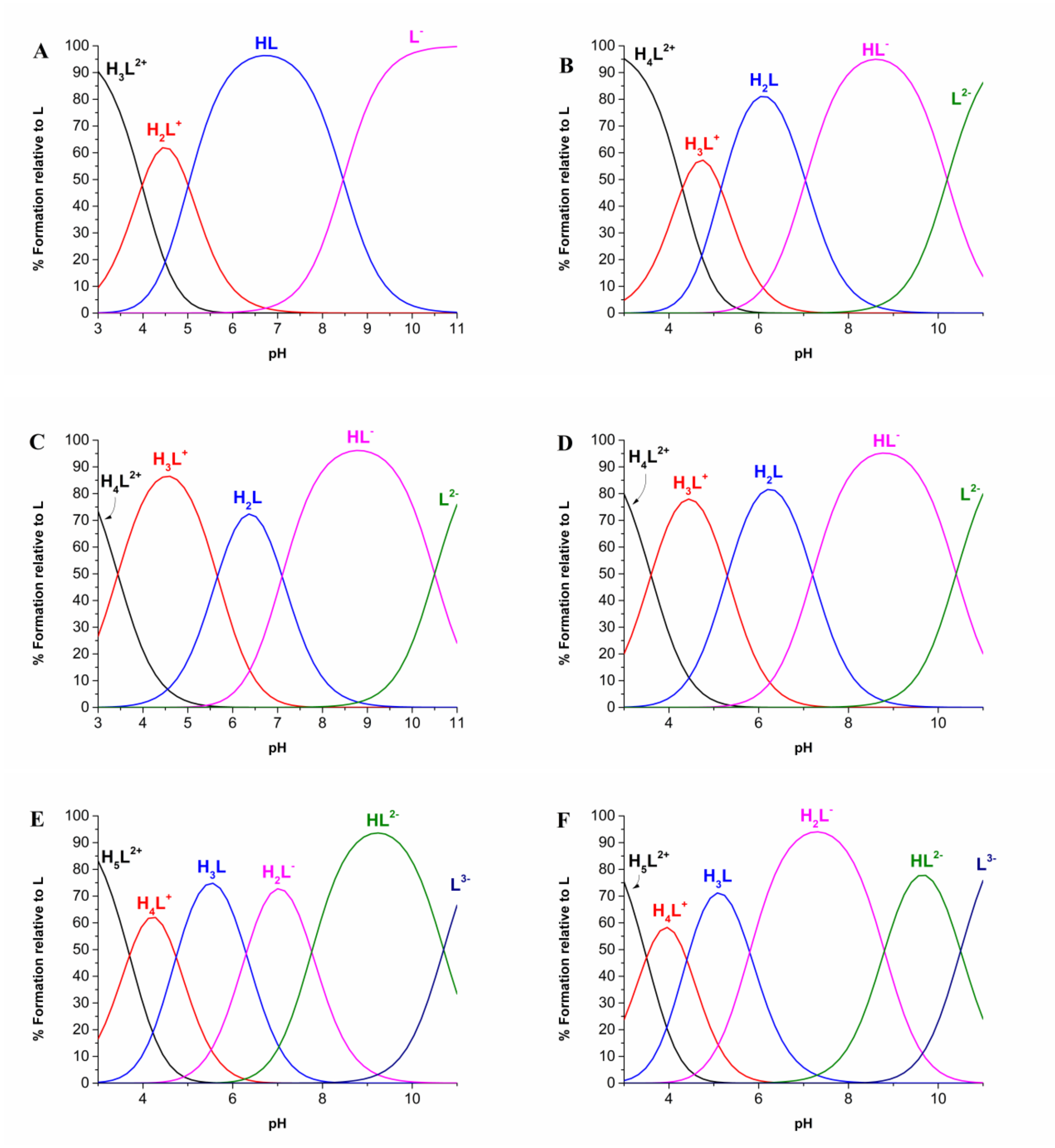

Figure S14 pH distribution curves for **T1-6** (**A-F**). **T1** $1.21 \cdot 10^{-4}$ M, **T2** $1.22 \cdot 10^{-4}$ M, **T3** $1.22 \cdot 10^{-4}$ M, **T4** $1.26 \cdot 10^{-4}$ M, **T5** $1.19 \cdot 10^{-4}$ M, and **T6** $1.19 \cdot 10^{-4}$ M. NaCl 0.1 M, 25 °C.

A

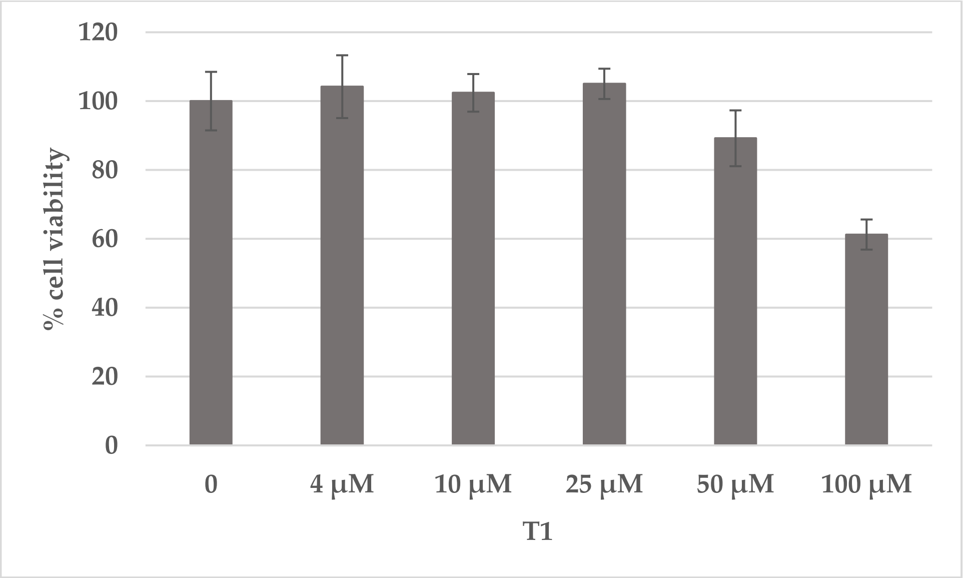

B

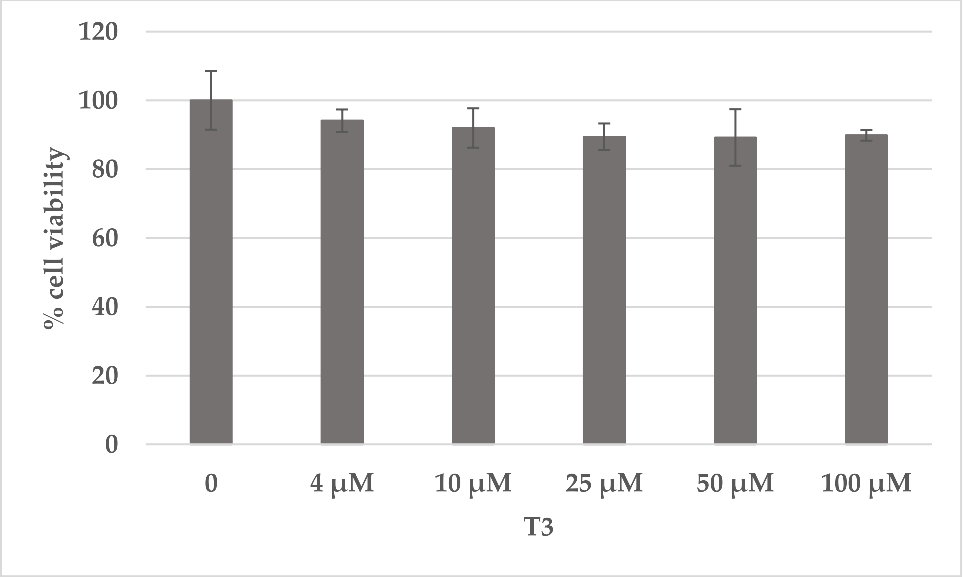

Figure S15. The effect of compound **T1** (**A**) and **T3** (**B**) on B16F10 cell at different concentrations (4–100 μM). Data are expressed as a percentage of the control. Data are presented as the mean ± the SD from three experiments.

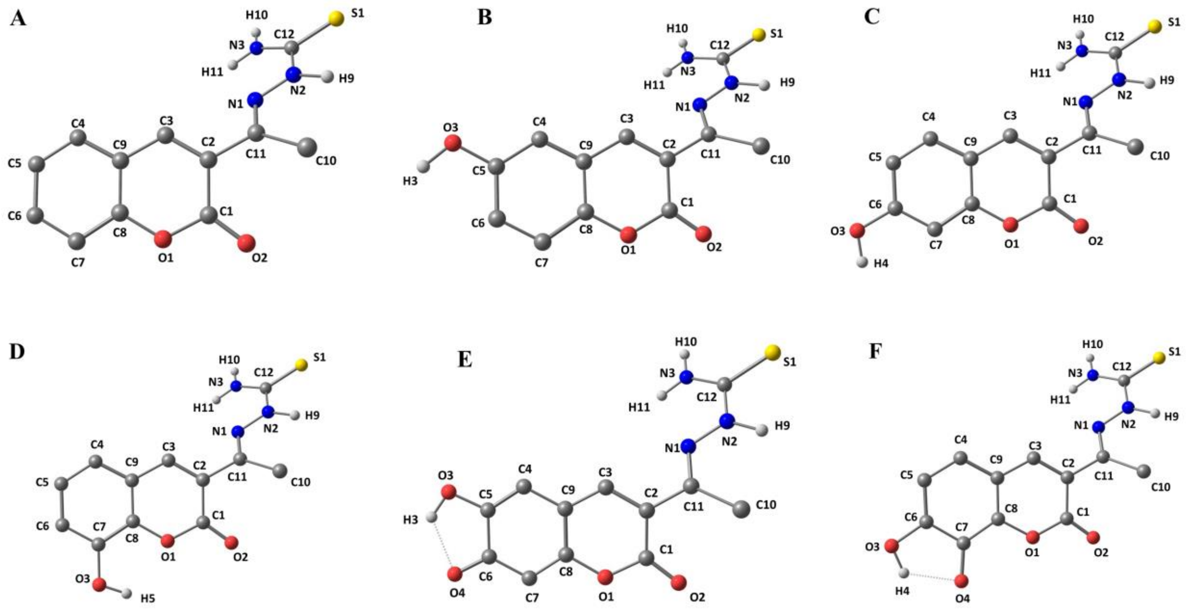

Figure S16. Molecular drawings and atom labelling scheme for the predominant species at pH 6.8 of **T1-6** (DFT: PBE0, def-2 TZVP, CPCM water).

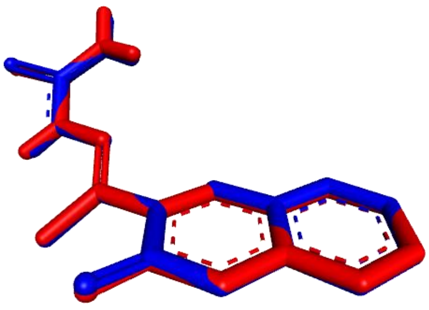

Figure S17. Superimposition of the X-Ray structure (blue coloured) and the DFT-optimized one (red coloured) of **T1**. The RMSD value obtained for the optimized structure was 0.12 Å.

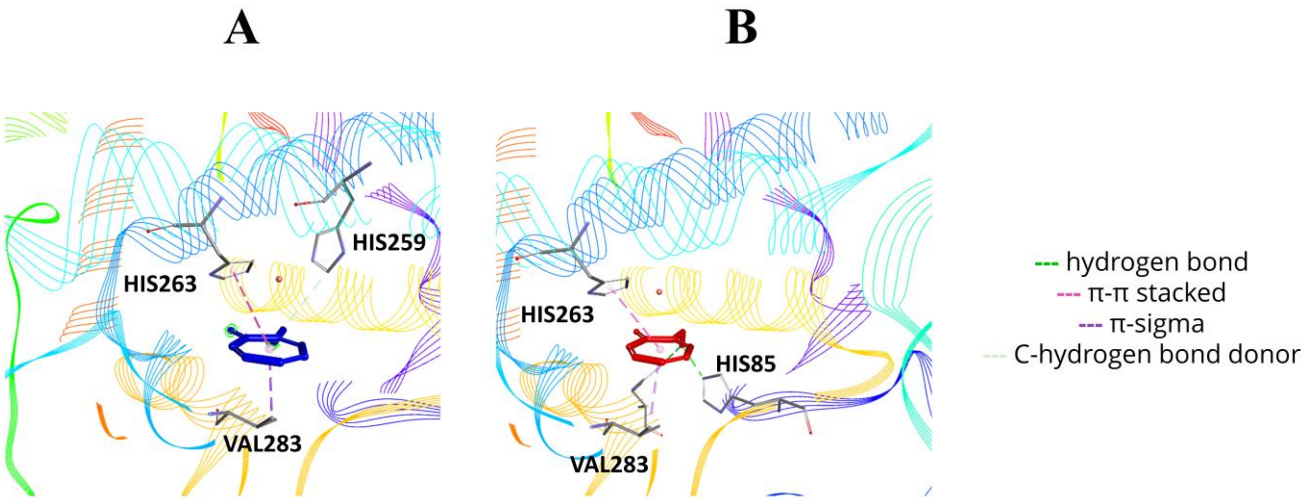

Figure S18. Intermolecular interaction profile between: (**A**) the X-Ray structure of OTR in the active site of mushroom tyrosinase; (**B**) the docked pose of OTR in the active site of mushroom tyrosinase.

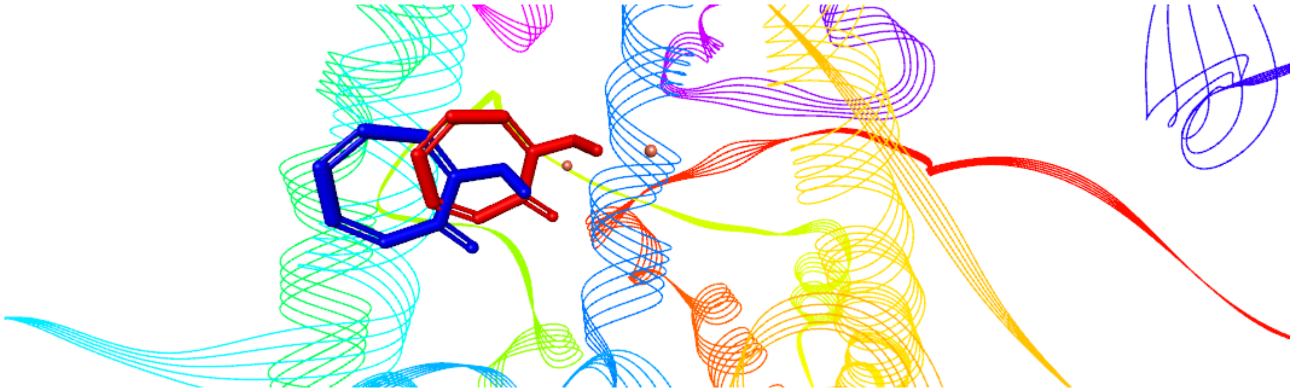

Figure S19. Superimposition of the crystallized pose (blue coloured) and the docked one (red coloured) of tropolone (OTR) in the active site of mushroom tyrosinase. The RMSD value obtained for the optimized structure was 2.32 Å.

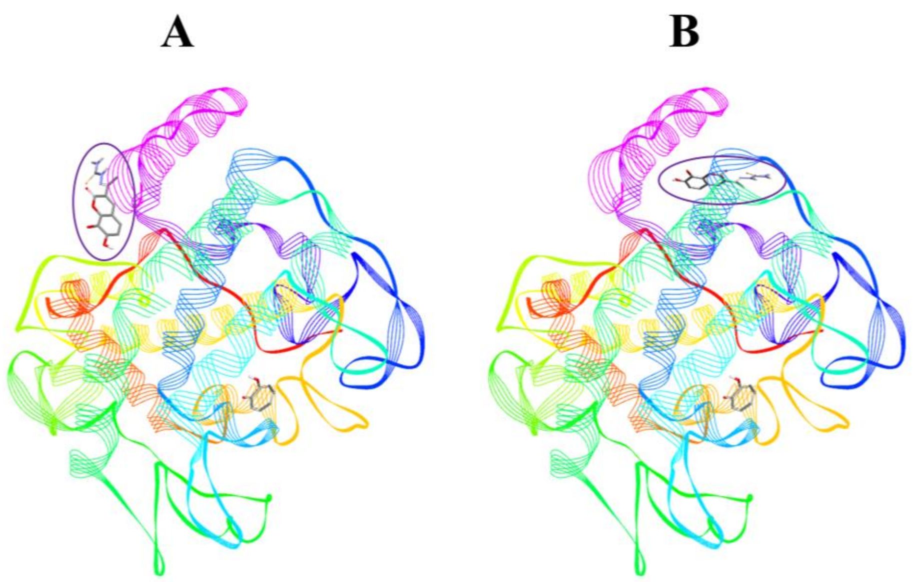

Figure S20. Docked pose of **T6** in allosteric MTb (**A**) and MTc (**B**) sites.

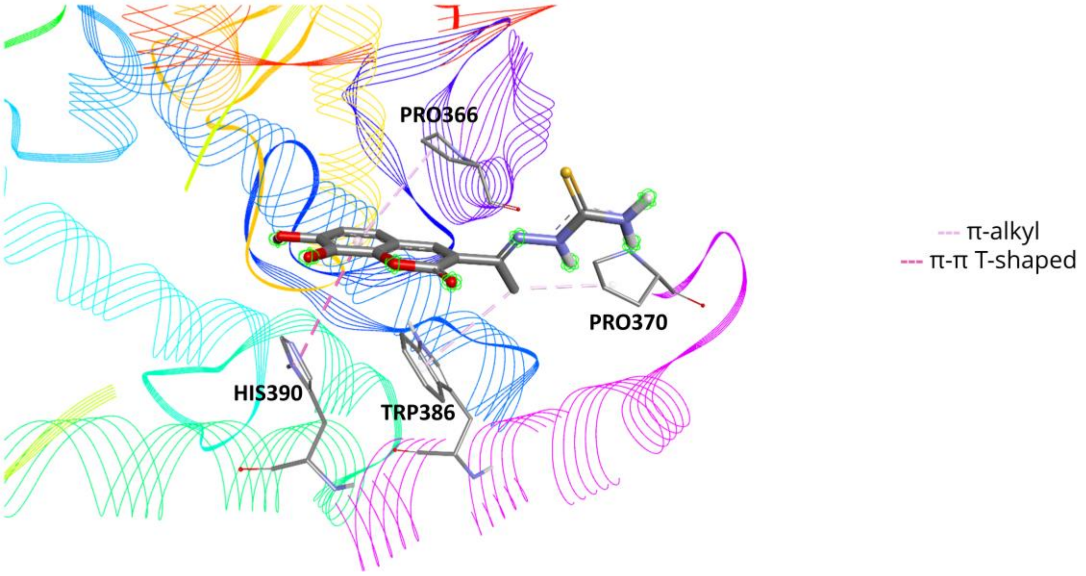

Figure S21. Docked pose of **T6** in the allosteric MTb site and intermolecular interactions with the surroundings residues of mushroom tyrosinase.

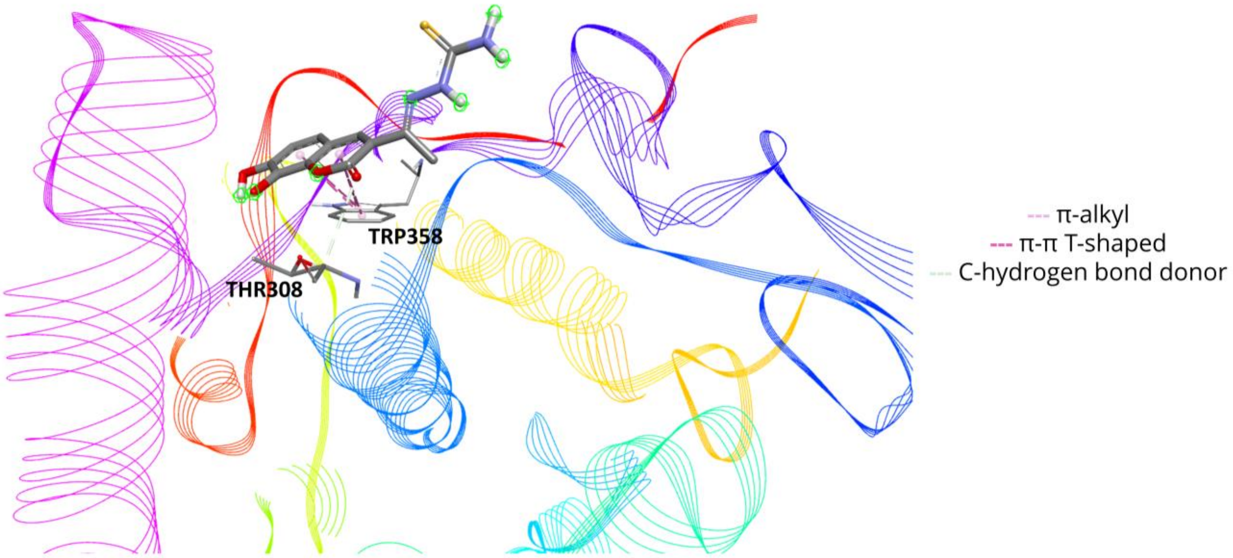

Figure S22. Docked pose of **T6** in the allosteric MTc site and intermolecular interactions with the surroundings residues of mushroom tyrosinase.

Table S1. Absorptivity values of the variously protonated species of T**1-6** (25 °C, NaCl 0.1 M).

Compound	Species	λ_{max} (nm)	ε(·10⁴ M⁻¹·cm⁻¹)
**T1**	L^{-}	270, 311 (sh)	1.04, 0.68
	HL	268, 330	1.26, 1.19
	H_2L^{+}	268, 332	1.32, 1.35
	H_3L^{2+}	268, 332	1.63, 161
**T2**	L^{2-}	271, 316 (sh), 406	2.22, 1.34, 0.85
	HL^{-}	271, 351	0.57, 0.28
	H_2L	269, 357 (sh)	0.48, 0.37
	H_3L^{+}	269, 348 (sh)	0.50, 0.33
	H_4L^{2+}	268, 350 (sh)	0.53, 0.37
**T3**	L^{2-}	263, 306, 429 (sh), 460	1.20, 0.95, 4.17, 0.46
	HL^{-}	273, 253	1.40, 2.17,
	H_2L	272, 350, 451	1.45, 2.32, 0.99
	H_3L^{+}	272, 351	1.45, 2.23
	H_4L^{2+}	272, 350	1.59, 2.42
**T4**	L^{2-}	275, 336	2.32, 1.15
	HL^{-}	263, 325	0.91, 0.96
	H_2L	263, 325	0.97, 1.00
	H_3L^{+}	265, 325	0.96, 1.00
	H_4L^{2+}	263, 325	1.14, 1.18

**T5**	L^{3-}	281, 316 (sh), 428, 475	1.35, 0.90, 2.71, 2.28
	HL^{2-}	269, 402	1.32, 1.48
	H_2L^{-}	265, 369	1.47, 2.11
	H_3L	266, 372	1.35, 1.72
	H_4L^{+}	267, 370	1.53, 2.01
	H_5L^{2+}	268, 371	1.40, 1.78
**T6**	L^{3-}	292, 382, 428	1.43, 1.25, 1.38
	HL^{2-}	270, 298 (sh), 442	1.09, 1.00, 2.06
	H_2L^{-}	275, 355	0.30, 0.68,
	H_3L	272, 442	0.68, 0.79
	H_4L^{+}	278, 355	0.38, 0.63
	H_5L^{2+}	272, 441	0.57, 0.78

Table S2. Selected optimized bond distances (Å) and angles (°) for DFT-optimized structure of **T1** (water) and corresponding X-Ray structural parameters. Atom labelling scheme as in **Figure S16A**.

	**T1**	
	Structural parameters	***DFT-optimized***
C1-O1	1.373	1.362
O1-C8	1.376	1.357
C1-O2	1.201	1.209
C11-N1	1.282	1.283
N1-N2	1.369	1.347
N1-C12	1.352	1.354
C12-S1	1.679	1.684
C12-N3	1.310	1.325
O1-C1-C2	116.10	116.83
C8-O1-C1	122.60	123.45
C1-C2-C11	118.51	118.83
C10-C11-N1	124.52	123.71
N1-N2-C12	119.10	120.50

N2-C12-N3	116.83	116.58
C8-O1-C1-C2	6.21	2.61
C1-C2-C11-C10	-42.11	-44.26
C10-C11-N1-N2	1.20	-0.32
C1-C2-C11-N1	141.10	138.85
C11-N1-N2-C12	175.22	178.68
N1-N2-C12-N3	4.70	-0.07

Table S3. Calculated Proton Affinity (PA) values for **T5** and **T6** (**H_3L**) compounds in gas phase and water at DFT level (PBE0, def-2 TZVP). Atom labelling scheme as in **Figure S16E-F**.

**COMPOUND**	**SITE**	**PA (Kcal/mol)**	
		**Gas phase**	**Water**
**T5**	H9	340.50	44.56
	H10/11	350.06	49.21
	H3	333.86	40.86
	H4	310.12	29.04
**T6**	H9	388.92	44.44
	H10/11	346.47	49.33
	H4	323.62	35.17
	H5	322.88	33.69
